# Optimization of Ultrasound-Assisted Obtention of Bluish Anthocyanin Extracts from Butterfly Pea (*Clitoria ternatea*) Petal Powders Using Natural Deep Eutectic Solvents

**DOI:** 10.3390/plants14071042

**Published:** 2025-03-27

**Authors:** Nicole Marina Almeida Maia, Irene Andressa, Jeferson Silva Cunha, Nataly de Almeida Costa, Larissa Lorrane Rodrigues Borges, Edimar Aparecida Filomeno Fontes, Eduardo Basílio de Oliveira, Bruno Ricardo de Castro Leite Júnior, Leonardo Lopes Bhering, Marleny Doris Aranda Saldaña, Érica Nascif Rufino Vieira

**Affiliations:** 1 Department of Food Science and Technology, Federal University of Viçosa, Viçosa 36570-900, MG, Brazil; nicole.maia0592@gmail.com (N.M.A.M.); irene.andressa@ufv.br (I.A.); natalyalmeida20@gmail.com (N.d.A.C.); larissa.borges@ufv.br (L.L.R.B.); eaffontes@ufv.br (E.A.F.F.); eduardo.basilio@ufv.br (E.B.d.O.); bruno.leitejr@ufv.br (B.R.d.C.L.J.); 2 Department of General Biology, Federal University of Viçosa, Viçosa 36570-900, MG, Brazil; leonardo.bhering@ufv.br; 3 Department of Agricultural, Food and Nutritional Science, Faculty of Agricultural, Life and Environmental Sciences, University of Alberta, Edmonton, AB T6G 2P5, Canada; marleny@ualberta.ca

**Keywords:** ultrasonic extraction, anthocyanins, green solvent

## Abstract

This study focused on improving the extraction of anthocyanins from medicinal plants using green solvents, which is important for the food, pharmaceuticals, and cosmetics industries. The goal was to optimize the time (15–50 min), temperature (40–80 °C), and petal/solvent ratio (2.5/7%) for the ultrasound-assisted extraction of anthocyanins from Butterfly Pea (*Clitoria ternatea*), using a natural deep eutectic solvent (choline chloride/glycerol, ChCl:Gly). The extraction was compared with a simple water extraction. To assess stability, we analyzed the anthocyanin content, antioxidant capacity, and color changes over 21 days. The optimal results were achieved using a temperature of 80 °C for 50 min and a 7% petal/solvent ratio. The CHCl:Gly solvent resulted in higher anthocyanin levels (374.65 mg DGE/L) compared to water (211.63 mg DGE/L). After storing the CHCl:Gly extract at 5 °C, only 16% of anthocyanins were lost, while the water extract lost 38%. The CHCl:Gly extract also showed better antioxidant capacity (156.43 µmol/mL). Color changes were less noticeable in the CHCl:Gly extract, especially when refrigerated. These findings demonstrate the method’s effectiveness for producing bioactive extracts, with potential for the food, pharmaceutical, and cosmetic industries.

## 1. Introduction

The use of edible flowers in food relates to cultural practices and is generally associated with beneficial effects on health. In addition to the aesthetic beauty, delicacy, and perception of freshness brought by flowers, edible flower extracts aim to improve the chemical properties of foods, such as their preservative and coloring potential and nutritional profile [1].

Butterfly Pea (*Clitoria ternatea*), commonly known as butterfly pea, belongs to the *Fabaceae* family and *Papilionaceae* subfamily, a perennial herbaceous plant in Indonesia. It can grow up to 2–3 m in many equatorial tropical areas [2], being adaptable to different temperature and humidity conditions [3]. It has been demonstrated that bioactive compounds extracted from the seeds, roots, flowers, and leaves of *Clitoria ternatea* have potential antioxidant [4], antimicrobial [5], and hepatoprotective activities [6]. In addition to the aforementioned properties, *Clitoria ternatea* flowers are widely used in cooking and the industrial sector due to the various anthocyanins that give them their blue color [2]. Ternatins, the predominant anthocyanins found in the flowers of *Clitoria ternatea*, are polyacylated compounds that, due to this structural modification, exhibit enhanced stability when exposed to light and fluctuations in pH, compared to non-acylated anthocyanins. This increased stability makes the plant extract highly promising for a wide range of applications in the food and pharmaceutical industries [7].

Concerning the techniques for extracting anthocyanins, liquid–solid extraction using organic solvents such as methanol and ethanol are amongst the most used methodologies [8]. However, these organic solvents are volatile and flammable, and their residues have been associated with some toxicity [9]. This combination of factors has led to the introduction of restrictive legislation, such as Directive 2010/75/EU issued by European Parliament, which aims to limit the use of these solvents in the pharmaceutical, cosmetic, and food industries [9]. On the other hand, an alternative group of solvents, called natural deep eutectic solvents (NADESs), are increasingly being explored to promote sustainable extraction following the basic principles of green chemistry [10]. These solvents are mixtures of naturally occurring eutectic substances such as sugars, amino acids, and organic acids that combine in specific molar proportions to reach the lowest melting point or eutectic point [11]. NADESs offer multiple benefits, including adjustable viscosity, biodegradability, simple separation, and customized extraction approaches [12].

In terms of processes, ultrasound-assisted extraction (UAE) technology has been widely and successfully explored in the extraction of natural compounds such as anthocyanins or other polyphenols [13,14,15,16,17]. This approach helps to optimize process yield and reduces nutritional and functional changes caused by the extracted compound [18]. UAE is based on the use of acoustic cavitation to promote the rupture of plant cell walls and vacuoles [13], which facilitates the penetration of the NADES and, consequently, improves the release, diffusion, and dissolution of target compounds [14]. Finally, a crucial aspect in extractions that combine UAE and NADESs to maximize the yield of the target compound is process optimization. The Box–Behnken method is commonly used to determine optimal extraction conditions to achieve the maximum yield of the extracted products. It is an experimental design that facilitates data collection while minimizing the number of experiments required [16,19].

Given these considerations, studies in this field have primarily focused on investigating the extraction of phytochemicals from *Clitoria ternatea* using conventional methods with aqueous solvents, such as ethanol or methanol [20,21,22]. However, to the best of our knowledge, there is a scarcity of studies exploring ultrasound-assisted extraction using a NADES. Thus, aiming to provide an original contribution in this field, this study aimed to optimize the ultrasound-assisted extraction of anthocyanins from *Clitoria ternatea* petals using the NADES choline chloride/glycerol, as well as evaluate the anthocyanin content, antioxidant capacity, and colorimetric stability of extracts under different storage conditions.

## 2. Materials and Methods

### 2.1. Plant Material

*Clitoria ternatea* flowers were purchased dehydrated (Flor Fada Azul, Mato Grosso do Sul, Brazil) with a water activity (*a_w_*) of 0.669 ± 0.003. The petals were separated and then dried in an oven with air circulation (Sinergia Científica^®^ SSDcr, Campinas, Brazil) for 12 h at 40 °C. After drying, the petals were grounded using a primary analysis mill (IKA^®^ A11 B S32, 180 W, São Paulo, Brazil) and sieved with an opening of 0.590 mm. The sieved petals were then classified based on the standard series of sieves. The powder (a_w_ = 0.488 ± 0.004) retained on the sieve with an average diameter of 28 mesh (Telastem Peneiras LTDA, São Paulo, Brazil) was used for the experiments. The samples were vacuum-sealed using a Cetro vacuum sealer (model CCVS 260 T, São Paulo, Brazil) in polyethylene bags and subsequently stored in a desiccator for 12 h before the analysis.

### 2.2. Preparation of the NADES

The NADES choline chloride/glycerol was chosen due to its low cost, high viscosity, and superior performance in extracting industrially relevant compounds compared to other types of NADESs [9]. The preparation of the NADES followed the protocol described by Chanioti and Tzia [23]. For this purpose, the solution was prepared by heating the two components at 80 °C under constant stirring until a homogeneous liquid was formed. Then, 30% (*v*/*v*) water was added, and the choline chloride–glycerol–water mixture was used in the extraction experiments, after being kept at −18 °C and protected from light.

### 2.3. Ultrasound-Assisted Extraction and Its Optimization

Powdered petals of *Clitoria ternatea* were subjected to ultrasound-assisted extraction of anthocyanins, using the deep natural eutectic solvent mixture of choline chloride/glycerol (1/2) with 30% water added (ChCl/Gly) or distilled water (control). The extractions were carried out in an ultrasonic bath (Unique, model USC 2800 A, Indaiatuba, Brazil) featuring heating control, a capacity of 9.5 L, a frequency of 40 kHz, a nominal power of 450 W, and a volumetric power of 38 W/L. The ultrasonic bath was filled with 9 L of distilled water, and the experiments were conducted individually in a random order to minimize experimental error, as outlined in Table 1. For each extraction, the experiments were performed at the maximum exposure point to ultrasonic intensity, as previously determined by the aluminum foil method [24].

For the extraction processes using ChCl/Gly, a Box–Behnken design (BBD) was employed to combine the extraction process variables, which included time (15, 32.5, or 50 min), temperature (40, 60, or 80 °C), and the solvent/powdered petal mass ratio at different concentrations (2%, 4.5%, or 7%) (Table 1).

Therefore, 15 combinations of the 3 variables at three levels, including 3 central points, were studied (Table 1). This aimed to identify the optimal process conditions of the combined controllable variables to extract total anthocyanins from *Clitoria ternatea* simultaneously. The study compared the extraction of total anthocyanins, antioxidant activity, and colorimetric stability in extractions with CHCl:Gly and water. The best process conditions of each of the controllable variables (80 °C, 60 min, 7%) were then simultaneously applied in the aqueous extraction of the petals.

### 2.4. Evaluation of Storage Stability of the Extracts Obtained Under Optimized Conditions

The stability of extracts of powdered *Clitoria ternatea* petals was assessed in terms of the total anthocyanin content, antioxidant capacity, and colorimetric values every 7 days during 21 days of storage, according to the methodologies detailed in the subsections hereafter.

For light stability, samples of powdered *Clitoria ternatea* petal extracts were placed in glass vials in a temperature-controlled room (27 ± 2 °C) and monitored until reaching 1800 lux, corresponding to daylight. Alternatively, some extracts were stored in a temperature-controlled room (27 ± 2 °C) and protected from light. For temperature, samples of powdered *Clitoria ternatea* petal extracts were placed in amber glass bottles, protected from light, in a temperature-controlled refrigerator (5 ± 2 °C), and the other samples remained in amber glass bottles in an incubator (TF-33A, Telga, Belo Horizonte, Brazil) with a temperature of 27 ± 2 °C.

#### 2.4.1. Determination of Total Anthocyanins

The total anthocyanin content was measured by spectrophotometry (Shimadzu^®^ UV-VIS 1800, Kyoto, Japan) using the single-pH method and the molar absorptivity coefficient of 29,000 L/mol·cm of delphinidin-3-glucoside. An aliquot of the samples was diluted in a solution of ethanol/HCl 1.5 M (85/15 *v*/*v*), with a reading at 535 nm, aiming to obtain an absorbance value between 0.200 and 0.800 [25]. Results were expressed as mg of delphinidin-3-glucoside (DGE) per L of extract (mg DGE/L). The total anthocyanin content of the diluted sample was calculated according to Equation (1).

(1) $$\mathrm{Total}\;\mathrm{anthocyanin}\;\mathrm{content}\;(\mathrm{mg}\;\mathrm{DGE}/L)=\frac{A\times MM\times FD\times 1000}{e\times 1}$$

In Equation (1), A is the absorbance measured at 535 nm, MM is the molecular weight of anthocyanin equivalent delphinidin-3-glucoside = 500.8 g/mol, dilution factor (FD) = 10, and the molar absorbance of delphinidin-3-glucoside e = 29,000 L/mol.cm. The anthocyanin content was calculated from the delphinidin-3-glucoside equivalent.

#### 2.4.2. DPPH Antioxidant Capacity

The trolox equivalent antioxidant capacity (TEAC) assay was conducted using the DPPH radical, with absorbance measurements taken at 517 nm (Shimadzu^®^ spectrophotometer model UV-VIS 1800, Kyoto, Japan). The results were expressed in µmol of Trolox per gram of sample [26].

#### 2.4.3. Objective Color Analysis and Total Color Difference (ΔE)

The instrumental color analyses of the extracts were performed using a colorimeter ColorQuest^®^ XE (Hunterlab, Reston, VA, USA) in reflectance mode and on the CIE Lab scale, using D65/10° illuminant. The coordinates lightness (L*), hue (h°), and saturation (C*) representing the color of a sample can also be expressed in cylindrical coordinates of lightness parameters, and were calculated from the measured a* and b* (Equations (2) and (3)). The total color difference (ΔE), Equation (4), was used to verify the colorimetric stability of extracts during the storage period under controlled light and temperature conditions.

(2) $$C^{*}=\sqrt{\left(a{*}^{2}+b*2\right)}$$

(3) $$h{}^{\circ}=\frac{b*}{a*}$$

(4) $$∆E=\sqrt{(∆L*)2+(∆a*)2+(∆b*)2}$$

### 2.5. Statistical Evaluation

RStudio^®^ software 4.3.0 was used to obtain the experimental Box–Behnken design adopted in the ultrasound-assisted extraction study. The experimental responses were fitted with a polynomial model, and analysis of variance (ANOVA) was applied to determine the linear, quadratic, and interacting regression coefficients of the model at a 95% significance level (*p* < 0.05). The model was evaluated by the coefficient of determination (R^2^), whereas the significance of the coefficients was analyzed by the *F*-value (*p* < 0.05).

The stability experiment was designed using a subdivided plot scheme, with a 2 × 4 factorial and the storage time in the subplot. Three replications produced 24 samples and 72 experimental units.

## 3. Results and Discussion

### 3.1. Optimization of the Ultrasound-Assisted Extraction Process of Anthocyanins Using the NADES

The results obtained from the experimental design are reported in Table 2. The total anthocyanin ranged from 12.95 to 85.48 mg DGE/L, and the predicted anthocyanin levels ranged from 12.97 to 82.94 mg DGE/L, showing that the independent variables (time, temperature, and powder/solvent ratio) considered in this study influenced the anthocyanin extraction process from *Clitoria ternatea*. Other studies that used UAE, as well as the combination of UAE and a NADES, also observed variations in anthocyanin levels in different plant matrices [27,28,29,30], reinforcing the importance of optimizing these processes.

The comparison between the experimental and predicted values showed an average relative percentage error of 21.8%. Pashazadeh et al. [31] reported differences between these values ranging from 13.7% to 17.8% when extracting anthocyanins from okra flowers, while Boateng et al. [32] extracted polyphenols from purple corn residue and observed differences of less than 10%. These variations may occur due to analytical interferences and changes in experimental conditions. However, the validation of the model is supported by the excellent reproducibility of the center point, where the experimental values were very close to the predicted values, representing an average relative percentage error of 5.6%.

The Shapiro–Wilk residual normality test (*p* < 0.05) presented a *p*-value = 0.9037, and the data for this analysis are from a normally distributed population. The linear effects of the temperature, time, and powder/solvent ratio were significant, as well as the interaction between the time and powder/solvent ratio (*p* < 0.05) (Table 3). The other interactions between the different pairs of factors and the quadratic terms were not significant (*p* > 0.05). Therefore, considering only the linear terms and a single interaction simplifies and accelerates result interpretation.

The ANOVA analysis used to validate the linear model fitted to experimental data also evaluated the influence of the factors on the response variables (Table 4). The coefficient of determination greater than 80% indicates that the Equation found describes the experimental values [33]. From this perspective, the coefficient of determination found was 83.36%, so the predicted and experimental anthocyanin content values fit well with the regression model.

ANOVA validated the predictive equation with the model fit, demonstrating a pure error lower than the F tab value. The lack of model fit was not significant (*p* > 0.05); therefore, the predicted equation was validated (Equation (7)). According to the adjusted equation, for the highest extraction of anthocyanins (Y_max_ = 93.85 mg DGE/L), the coded values of the variables X_1_, X_2_, and X_3_ corresponded to the maximum coded points (+1).

(5) $$Y=40.88687+(13.27552*X_{1})+(10.90853*X_{2})+(17.00963*X_{3})+\left(11.7645*X_{1}*X_{3}\right)$$

In Equation (5), Y is the total anthocyanin content (mg DGE/L), X_1_ is the temperature, X_2_ is the time, and X_3_ is the powder/solvent mass ratio.

Temperature is a critical factor in the extraction of anthocyanins, as these compounds are thermolabile, and their stability can be compromised at unsuitable temperatures [34]. In studies using conventional solvents such as 60% ethanol, Santos and Martins [27] determined that the optimal temperature for the ultrasonic extraction of anthocyanins from *Clitoria ternatea* was 45 °C. However, in the present study, the predictive model indicated an optimal temperature of 80 °C (variable X_1_) for extracting these compounds.

**Figure 1:**
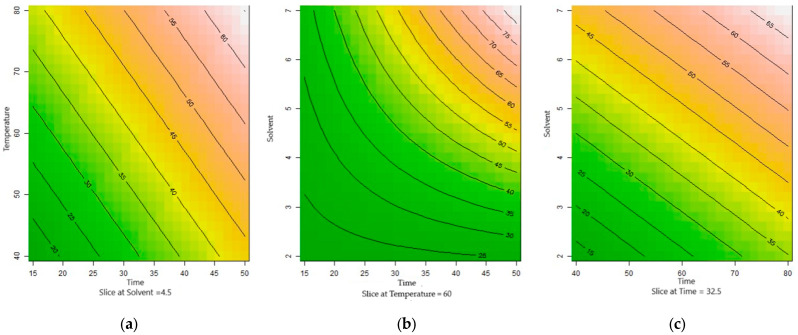
Contour graph of 2^2^ combinations with the cut at the central points (0). (**a**) Linear effect of temperature on anthocyanin extraction, with time fixed at 32.5 min and powder/solvent ratio at 4.5%. Higher temperature increases anthocyanin extraction. (**b**) Interaction between time and powder/solvent ratio on anthocyanin extraction, with temperature fixed at 60 °C. (**c**) Linear effect of temperature and solvent on anthocyanin extraction, with time fixed at 32.5 min and powder/solvent ratio at 4.5%.

At lower temperatures, the viscosity of the NADES was high (>100 cP) due to van der Waals interactions between its components [35]. For efficient extraction with the CHCl:Gly solvent, the solvent must easily penetrate the pores of the solid matrix, which is facilitated by the reduction in viscosity at higher temperatures. Therefore, the elevated temperature did not hinder the extraction of anthocyanins using the NADES mixture. It is important to note that NADESs typically have a relatively high decomposition temperature, meaning that the extraction temperature must stay below the decomposition point of the eutectic mixture to optimize the performance of these alternative solvents [36]. Thus, the observed trend of increased anthocyanin extraction with rising temperature (Figure 1a) in the present study is consistent with previous findings in the literature.

The variable X_2_ (time) indicated that 50 min is an efficient time to extract anthocyanins from *Clitoria ternatea* powder using CHCl:Gly solvent. However, an exposition time of 50 min may degrade or extract fewer anthocyanins using water. This is because prolonged exposure to ultrasonic waves promotes the disruption of the cell wall and vacuoles and allows the permeation of the solvent and the diffusion of compounds when using water [37]. The increase in the internal energy of the system caused by long extraction processes can lead to the degradation of several compounds, particularly the structure of the central ring of anthocyanins, generating unstable oxidized compounds (anhydrous compounds and chalcones) [27].

The variable X_3_ indicated that a powder/solvent ratio of 7% provides the best total anthocyanin content. NADESs have high viscosity due to robust hydrogen interactions, resulting in lower molecule mobility [38]. This statement justifies the interaction between variables X_1_ and X_3_ in the model. This positive correlation, previously observed in other studies [39], indicates that the temperature reduced the solvent’s viscosity and increased the mobility of the molecules, leading to better extraction of the compounds [40].

The linear polynomial model mathematically represents the effects of temperature, time, and the powder/solvent ratio and their interactions on the extraction of anthocyanins from powdered *Clitoria ternatea* petals at the central point of the experiment (Figure 1). The linear effect that time and temperature have on the response variable with the main point is fixed on the powder/solvent ratio (4.5%), that is, the higher the temperature, the greater the extraction of anthocyanins (Figure 1a). In Figure 1c, the linear effect on the solvent and temperature variables with the central point in time is visible (32.5 min). Figure 1b shows the extraction effect of the interaction of the time and powder/solvent ratio variables, with a central point fixed at temperature (60 °C). The optimization of anthocyanin extraction from *Clitoria ternatea* has been the subject of a few studies, each using different approaches regarding process parameters and extraction yields. Pham et al. [20] optimized anthocyanin extraction using 50° ethanol as a solvent, achieving 132.756 mg DGE/L of total anthocyanins at a solid/liquid ratio of 23/1, an extraction time of 46 min, and a temperature of 60.6 °C. In contrast, Santos and Martins [27] found optimal extraction conditions at 45 °C for 30 min, using 300 W of ultrasonic power, which resulted in 343.18 equivalents of cyanidin-3-glucoside/100 g with ethanol as the solvent. These results differ from each other and the optimal conditions identified in the present study, likely due to differences in the solvent used and the ultrasonic power applied.

Regarding anthocyanin content, both studies reported higher yields than this study (93.85 mg DGE/L). However, it is important to note that these studies did not evaluate the stability of the compounds during storage. The stability of anthocyanins is a critical factor; it is not only the extraction yield that matters but also how these compounds behave over time, influenced by factors such as temperature and oxidation.

Recent studies suggest that anthocyanins extracted with NADESs exhibit greater stability during storage compared to traditional solvents like water, ethanol, and methanol [41]. The hydrogen bonds in these systems help make the molecules more resistant to hydrolysis and oxidative degradation, which are key factors that compromise anthocyanin stability. High temperatures and the presence of oxygen accelerate the degradation of these compounds, emphasizing the importance of solvent choice and extraction conditions to ensure the preservation of the antioxidant properties of anthocyanins over time [42].

### 3.2. Evaluation of Stability

#### 3.2.1. Objective Color Analysis and Total Color Difference (ΔE)

**Figure 2:**
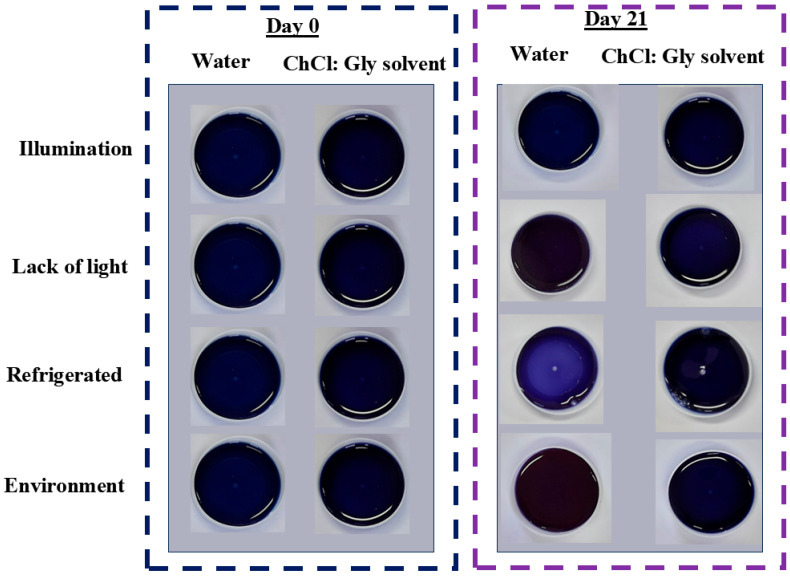
Photographic records of extracts on days 0 and 21 of storage under different storage conditions.

Photographs of the extracts were recorded on the day of extraction (day 0) and the 21st day of storage (Figure 2). On day 0, the extracts had the same bluish color. However, the visual records taken on day 21 showed that aqueous extracts acquired different colors, whereas CHCl:Gly extracts presented homogeneity in their color. This result is further supported by the lower loss of antioxidant activity and total anthocyanins in the samples extracted with ChCl/Gly. This can be attributed mainly to the hydrogen interactions between the solvent and these compounds. These interactions help inhibit oxidation reactions, which are responsible for anthocyanin degradation, thereby preserving the blue coloration of the extracts.

Table 5 and Figure 2 represent the color instrumental attributes of L*, C*, h*, and overall color variation (∆E) found for extracts obtained with water and ChCl:Gly under different storage conditions for 21 days.

Aqueous extracts were clear and had a higher luminosity (L*) attribute when compared to extracts with CHCl:Gly (*p* < 0.05). The ΔE values were higher for aqueous extracts than those obtained using CHCl:Gly as extraction solvent, corroborating a more significant color variation in aqueous extracts (Table 5). Data in Figure 2 show that this behavior prevailed during 21 days of storage under different conditions (*p* < 0.05).

Based on the overall color variation (∆E) over the storage period, more significant changes were observed in the aqueous extracts across all four storage conditions, with less pronounced alterations when stored under refrigeration or at room temperature (Figure 2). Up to 14 days of storage, the color differences between the samples compared to day 1 were not perceptible to the human eye, as the ∆E remained below 2 [43]. However, after 21 days of storage, all aqueous extracts, except those stored under refrigeration, displayed perceptible differences to the human eye (∆E > 2), as evidenced by Figure 2. For the extracts obtained with ChCl/Gly, only those stored under light or at room temperature showed noticeable color variations compared to the first day. Figure 2 and Table 5 support these findings, as the samples with the greatest reduction in anthocyanin content also exhibited the most significant instrumental color changes by the end of the storage period. This was attributed to the degradation of the anthocyanins responsible for the blue coloration, which was less pronounced in the CHCl:Gly extracts when compared to the aqueous extracts.

The aqueous extracts exhibited a 9.8% change in luminosity (L* value) after 21 days of storage without light protection, indicating a slight increase in lightness (*p* < 0.05). Conversely, when protected from light, there was no significant change in luminosity (*p* < 0.05). For the CHCl:Gly extracts, luminosity changed by 2.5% under ambient conditions and 12.0% under light exposure. Overall, storage conditions slightly influenced the luminosity of both extract types.

**Figure 3:**
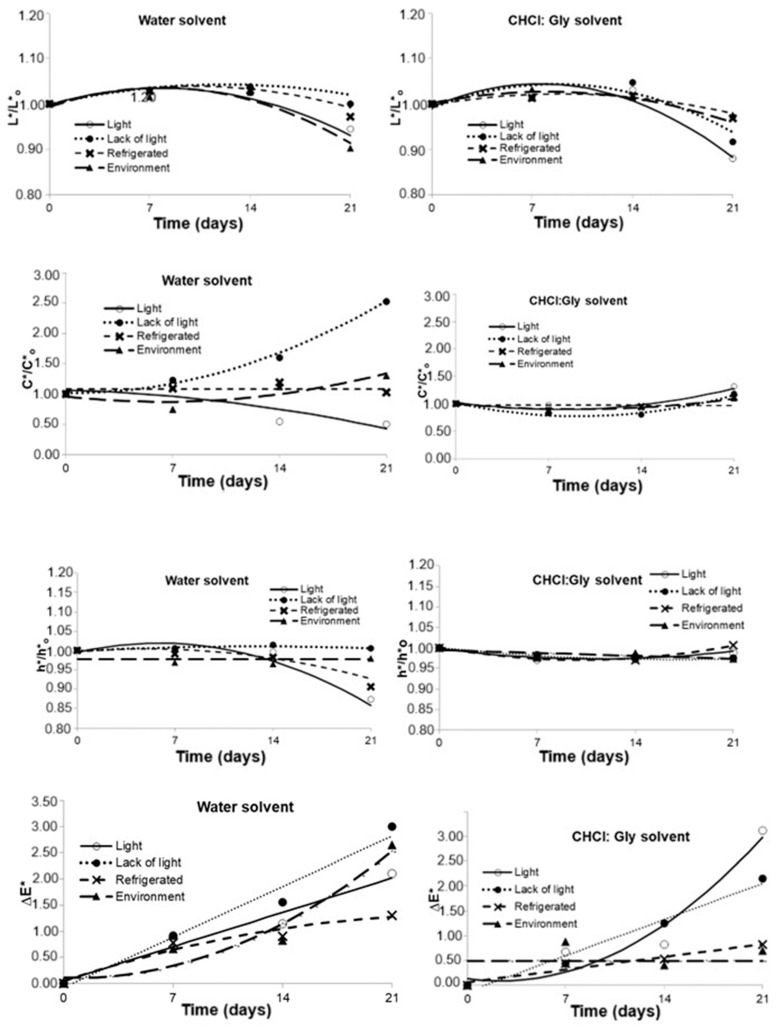
Variation of color attributes of powdered Butterfly Pea (*Clitoria ternatea*) petal extracts using water and ChCl:Gly under different storage conditions for 21 days.

Regarding tonality (h* value), as shown in Figure 3, the CHCl:Gly extracts exhibited minimal variation between storage conditions, with a slight increase in tonality of 0.6% under refrigeration and a decrease of 2.6% under ambient conditions after 21 days. Consequently, the tonality was only marginally affected by the type of extracting solvent.

For saturation (c* value), the aqueous extracts showed a 152.7% increase under light protection, while a 49.8% decrease was observed under light exposure. In contrast, the variations in saturation for CHCl:Gly extracts were smaller, with a 31.6% increase under light exposure and a 9.6% increase under ambient conditions after 21 days. Furthermore, color analysis using the full set of L*, a*, and b* parameters provides a more comprehensive assessment than analyzing each parameter in isolation [44].

The key areas of food technology consider the study of color a fundamental aspect of product appearance. Consumers often do not assess factors such as taste and texture if a food product does not appear appealing. The study of colorimetric coordinates is an excellent tool for evaluating food quality, as it enables monitoring changes that may indicate variations in processing, storage, or control. In addition to instrumental color, the pH of the medium is another factor that can influence the stability of the extract color. The violet-blue coloration of *Clitoria ternatea* extract occurs within a wide pH range of 3.21 to 7.19 [45]. The violet color is specifically observed between pH 3 and 5 when the red flavil cation and two neutral blue quinonoid species tautomers are in equilibrium. During storage, color variation and pH are crucial for maintaining the visual and sensory properties of the product. The color stability throughout shelf life is directly related to maintaining the ideal pH, as changes in this variable can affect the extract’s hue and consumer perception. However, this variable was not evaluated in the present study despite its importance. Future research may include the evaluation of pH during storage to understand better its impact on anthocyanin degradation and the color stability of extracts obtained using NADESs.

#### 3.2.2. Total Anthocyanin Content and Antioxidant Capacity

**Figure 4:**
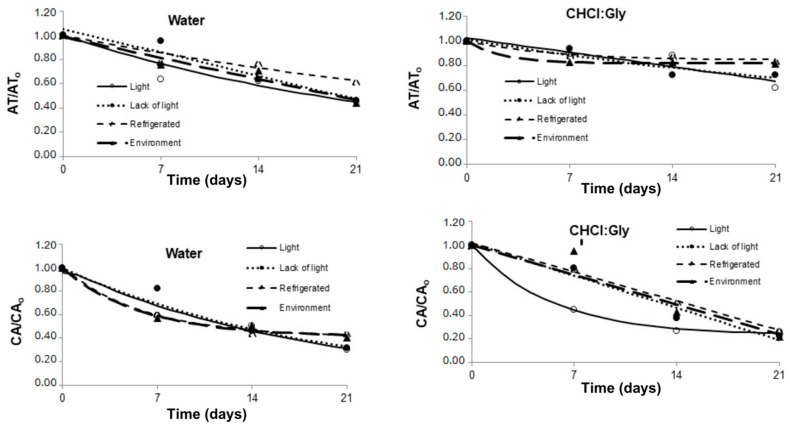
Variation of total anthocyanins and antioxidant capacity of powdered Butterfly Pea (*Clitoria ternatea*) petal extracts using water and CHCl:Gly solvents under different storage conditions for 21 days.

Table 6 and Figure 4 represent the total anthocyanin content (equivalent to delphinidin-3-glucoside; DGE mg DGE/L) and antioxidant capacity (in µmol of Trolox equivalent/mL) extracted using solvent water and CHCl:Gly under different storage conditions for 21 days.

The average values of the extracts prepared with CHCl:Gly exhibited a higher anthocyanin concentration (374.65 DGE mg DGE/L) than the aqueous extracts (211.63 DGE mg DGE/L). These results confirm that, even under the same extraction conditions, using CHCl:Gly as a solvent led to a significantly higher yield of total anthocyanins. Furthermore, when using water, a significant change in the anthocyanins was observed over the 21-day storage period, compared to the systems using CHCl:Gly (*p* < 0.05). The concentration of anthocyanins in the water decreased, although the loss was less pronounced when stored under refrigeration. In the former case, around 38% of the total anthocyanin content was lost early on, while the loss was only 16.3% in the refrigerated condition. These results suggest that refrigeration (5 ± 2 °C) appears more suitable for storing the extracts, while light can degrade the anthocyanins extracted by both solvents. Previous studies have also demonstrated the protective effect of NADESs on anthocyanins during storage, which can mainly be attributed to the hydrogen bonds between the solvent and the anthocyanins [46,47,48]. These bonds help protect the compounds against oxidation reactions, which are the primary cause of anthocyanin degradation over time [16].

The stability of anthocyanins is crucial for their application as natural colorants and for their beneficial health properties. However, their bioavailability can be influenced by several factors. The intermolecular interaction between the NADES and the anthocyanins is primarily responsible for their stability. This is due to the protective effect of weak hydrophobic intermolecular interactions, such as π-stacking involving the aromatic rings, against degradation reactions [45]. In this way, π-stacking reduces the susceptibility of these molecules to degradation by inhibiting the absorption of ultraviolet or visible light, which typically accelerates the breakdown of these compounds [49]. Another crucial factor in the stability of anthocyanins is the extraction temperature. According to Arruda et al. [50], the hydrolysis of the 3-glycosidic bond of anthocyanins occurs only at temperatures above 60 °C. Furthermore, the extraction temperature of 80 °C using CHCl:Gly was efficient because the stability of the anthocyanins is due to the strong hydrogen bonding interactions between target metabolites, CHCl:Gly molecules, and water, leading to a space structure that further holds the anthocyanins and protects them from environmental factors [51].

Light is another factor contributing to the degradation of anthocyanins. According to Patras et al. [52], the degradation of anthocyanin molecules can occur through the action of light and is enhanced by the presence of oxygen. In this sense, treatments in which the material was exposed to light had significant anthocyanin losses due to the photodegradation of their molecules. However, it is reassuring to note that the anthocyanins in the CHCl:Gly extracts were better preserved when subjected to light than their counterparts in aqueous extracts. This is due to the lower water activity of the solvent, which stabilizes the anthocyanins. Given the lower degradation of anthocyanins, one can hypothesize that intermolecular interactions exerted a protective effect against photodegradation, highlighting the role of the CHCl:Gly solvent in maintaining the quality of the extracts. In our present study, the best conditions for removing and storing the anthocyanins of *Clitoria ternatea* powder extracts were found to be extraction with the CHCl:Gly solvent stored at refrigerated temperatures (5 °C) and without light.

The mean values of the extracts prepared with the CHCl:Gly solvent showed a greater antioxidant capacity (156.43 µmol/mL) than those observed for the aqueous extracts (93.01 µmol/mL). During storage, antioxidant activity declined less in the CHCl:Gly extracts compared to the aqueous samples over the 21 days of storage under various conditions. This suggests that the hydrogen bonds formed between the solvent and the anthocyanins played a crucial role in reducing the loss of antioxidant activity during storage. Moreover, the total amount of anthocyanins extracted using CHCl:Gly was higher than that of the alcoholic extract, further reinforcing this finding. This trend has been observed in other studies comparing the stability of anthocyanins extracted with NADESs to those extracted with alcoholic and aqueous solvents [16].

Refrigeration proved to be the condition that best preserved antioxidant activity in both the aqueous extract and CHCl:Gly (Table 5). This outcome can be attributed to the high viscosity of CHCl:Gly under refrigeration, which slows molecular movement, promoting more stable molecular interactions between the solvent and anthocyanins, further enhancing the stability of these compounds [53]. Although aqueous extracts of anthocyanins can be freeze-dried to increase their stability, extracts obtained with NADESs have advantages in terms of efficient extraction and greater solubility of bioactive compounds, although with less flexibility for conversion into powder. Future work may explore strategies to improve the stability of NADES extracts, such as their incorporation into encapsulation systems or combination with drying techniques, such as spray drying. Furthermore, investigations into the application of these extracts in food and cosmetic matrices may expand their industrial possibilities, ensuring greater use of the bioactive compounds of *Clitoria ternatea*.

## 4. Conclusions

The present study improved the removal of anthocyanins from *Clitoria ternatea* powder using green solvents. The optimal results were achieved by using a temperature of 80 °C for a duration of 50 min and a 7% petal/solvent ratio, where the natural deep eutectic solvent (choline chloride/glycerol, ChCl/Gly) obtained higher anthocyanin levels (374.65 mg DGE/L) compared to water (211.63 mg DGE/L). During storage, all treatments showed anthocyanin losses, changes in antioxidant capacity, and total color difference. The stability of the blue color during refrigerated storage, with only 16% of anthocyanin turnover in 21 days, highlights the potential of these extracts for industrial applications. The extracts showed high color stability and antioxidant properties, confirming that the optimization of the ultrasonic removal process of *Clitoria ternatea* powder using the CHCl:Gly green solvent is a viable, sustainable, and efficient alternative for obtaining high-quality natural extracts.

Furthermore, the cost/benefit ratio of using NADESs is superior when compared to traditional solvents, such as water, methanol, and ethanol, due to the greater stability of anthocyanins extracted with NADESs throughout their useful life, making it a more viable option for applications such as in the food industry as natural dyes and pH indicators. Although the study demonstrated the effectiveness of the extraction, future research is essential to evaluate other process variables in addition to time, temperature, and the powder/solvent ratio, in addition to analyzing the stability of the extract for more than 21 days and reproducing long-term commercial applications. Therefore, future studies should consider these aspects to validate and expand the use of this technology.

## Figures and Tables

**Table 1:** Experimental model of the levels of selected variables.

Variable	Factor		Level	
	X	Low (−1)	Medium (0)	High (+1)
Time (min)	X_1_	15	32.5	50
Temperature (°C)	X_2_	40	60	80
Petal/solvent mass ratio (m/v)	X_3_	2%	4.5%	7%

**Table 2:** Box–Behnken design for independent variables of time, temperature, and powder/solvent ratio, and experimental and response total anthocyanin content (TAC).

Run	X_1_ (°C)	X_2_ (min)	X_3_ (%)	Time (min)	Temperature (°C)	Powder/ Solvent (%)	TAC Response (mg DGE/L)	TAC Predicted (mg DGE/L)
1	−1	−1	0	15	40	4.5%	23.31	16.70
2	+1	−1	0	50	40	4.5%	40.80	43.25
3	−1	+1	0	15	80	4.5%	49.54	38.52
4	+1	+1	0	50	80	4.5%	51.48	65.07
5	−1	0	−1	15	60	2%	12.95	22.37
6	+1	0	−1	50	60	2%	32.81	25.39
7	−1	0	+1	15	60	7%	18.56	32.86
8	+1	0	+1	50	60	7%	85.48	82.94
9	0	−1	−1	32.5	40	2%	19.64	12.97
10	0	+1	−1	32.5	80	2%	29.31	34.79
11	0	−1	+1	32.5	40	7%	43.04	46.99
12	0	+1	+1	32.5	80	7%	83.72	68.81
13	0	0	0	32.5	60	4.5%	41.28	40.89
14	0	0	0	32.5	60	4.5%	37.45	40.89
15	0	0	0	32.5	60	4.5%	43.93	40.89

X_1_: temperature; X_2_: time; X_3_: powder/solvent ratio; TAC: total anthocyanin content; DGE: equivalent of delphinidin-3-glucoside.

**Table 3:** Analysis of the isolated and combined effects of the independent variables of the extraction process.

Variables	Estimate	Std. Error	t-Value	*p*-Value
Intercept	40.8869	6.6250	6.1717	0.0016
X_1_ (L)	13.2755	4.0569	3.2723	0.0221
X_2_ (L)	10.9085	4.0569	2.6889	0.0433
X_3_ (L)	17.0096	4.0569	4.1927	0.0086
X_1_ * X_2_	−3.8855	5.7374	−0.6772	0.5283
X_1_ * X_3_	11.7645	5.7374	2.0505	0.0955
X_2_ * X_3_	7.7560	5.7374	1.3518	0.2344
X_12_ (Q)	−3.0381	5.9717	−0.5087	0.6326
X_22_ (Q)	3.4348	5.9717	0.5752	0.5901
X_32_ (Q)	−0.3968	5.9717	−0.0664	0.9496
Intercept	40.8869	2.6383	15.4974	2.554 × 10^−8^
X_1_ (L)	13.2755	3.6127	3.6747	0.0043
X_2_ (L)	10.9085	3.6127	3.0195	0.0120
X_3_ (L)	17.0096	3.6127	4.7083	0.0008
X_1_ * X_3_	11.7645	5.1091	2.3027	0.0441

* Statistical significance *p* < 0.05; X_1_: time; X_2_: temperature; X_3_: powder/solvent mass ratio; L: linear effect; Q: quadratic effect.

**Table 4:** Analysis of variance (ANOVA) of the linear model to extract anthocyanin content.

	DF	SS	MS	*F*-Value	*p*-Value
X_1_; X_2_; X_3_ (L)	3	4676.50	1558.83	14.9299	0.0005
X * X_3_	1	553.60	553.61	5.3023	0.0441
Residuals	10	1044.10	104.41		
Lack of fit	8	1022.90	127.86	12.0712	0.0787
Pure error	2	21.20	10.59		
R^2^ = 0.8336					

* Statistical significance *p* < 0.05; SS: sum of squares; DF: degrees of freedom; MS: mean square; X_1_: time; X_2_: temperature; X_3_: powder/solvent mass ratio; L: linear effect.

**Table 5:** Mean values standard deviation for color attribute values using ChCl:Gly and solvent water under different storage conditions for 21 days.

Factors/Interactions	Levels	Color Attribute		*p* > F ^1^	Color Attribute		*p* > F ^1^
		L* value			c* value		
Extracting solvent	ChCl/Gly	25.55 ± 1.09 ^a^		<0.0001	1.36 ± 0.18 ^a^		<0.0001
	Water	26.25 ± 0.91 ^b^			2.13 ± 0.90 ^b^		
Storage conditions	Illumination	25.77 ± 1.37 ^c^		<0.0001	1.53 ± 0.48 ^c^		<0.0001
	Lack of light	25.93 ± 1.13 ^b^			2.25 ± 1.30 ^a^		
	Refrigerated	26.00 ± 0.67 ^a^			1.66 ± 0.34 ^b^		
	Environment	25.90 ± 1.02 ^b^			1.53 ± 0.35 ^c^		
Extracting solvent * Storage conditions		ChCl/Gly	Water solvent	<0.0001	ChCl:Gly	Water solvent	<0.0001
	Illumination	25.37 ± 1.64 ^B,c^	26.18 ± 0.93 ^A,c^		1.39 ± 0.24 ^B^	1.67 ± 0.62 ^A,d^	
	Lack of light	25.31 ± 1.27 ^B,c^	26.54 ± 0.46 ^A,a^		1.38 ± 0.24 ^B^	3.12 ± 1.20 ^A,a^	
	Refrigerated	25.68 ± 0.52 ^B,b^	26.33 ± 0.66 ^A,b^		1.36 ± 0.13 ^B^	1.95 ± 0.16 ^A,b^	
	Environment	25.84 ± 0.56 ^B,a^	25.96 ± 1.36 ^A,d^		1.30 ± 0.12 ^B^	1.77 ± 0.36 ^A,c^	
		h* value, in degrees			∆E		
Extracting solvent	ChCl/Gly	343.33 ± 4.42 ^a^		<0.0001	0.76 ± 0.82 ^b^		<0.0001
	Water	346.50 ± 13.52 ^b^			1.05 ± 0.91 ^a^		
Storage conditions	Illumination	342.71 ± 14.4 ^b^		<0.0001	1.09 ± 1.01 ^a^		<0.0001
	Lack of light	350.08 ± 6.90 ^a^			1.16 ± 1.01 ^a^		
	Refrigerated	343.75 ± 10.4 ^b^			0.60 ± 0.46 ^c^		
	Environment	343.11 ± 4.77 ^b^			0.76 ± 0.81 ^b^		
Extracting solvent * Storage conditions		CHCl:Gly solvent	Water solvent	<0.0001	ChCl/Gly	Water solvent	<0.0001
	Illumination	342.20 ± 4.24	343.21 ± 20.4 ^b^		1.15 ± 1.23 ^A,a^	1.03 ± 0.78 ^B,b^	
	Lack of light	344.07 ± 4.10 ^B^	356.08 ± 1.99 ^A,a^		0.96 ± 0.86 ^B,b^	1.37 ± 1.15 ^A,a^	
	Refrigerated	344.22 ± 5.57	343.28 ± 13.9 ^b^		0.45 ± 0.35 ^B,c^	0.75 ± 0.53 ^A,c^	
	Environment	342.81 ± 3.79	343.41 ± 5.74 ^b^		0.49 ± 0.35 ^B,c^	1.03 ± 1.04 ^A,b^	

^1^ Significant probabilities (*p* < 0.05) by F test. For independent factors, means followed by different lowercase letters in the column differ using the t or Tukey’s tests (*p* < 0.05). In the interaction, for the same storage condition, in the row, means followed by different capital letters differ from each other using the *t*-test (*p* < 0.05). The means followed by different lowercase letters in the column for the same extracting solvent differ using Tukey’s test (*p* < 0.05).

**Table 6:** Mean values ± standard deviation for measuring antioxidant capacity and total anthocyanin (TAC) content extracted using CHCl:Gly and solvent water under different storage conditions for 21 days.

Factors/Interactions	Levels	Antioxidant Capacity (µmol of Trolox Equivalent/mL Extract)		*p* > F ^1^	TAC (mg DGE/L)		*p* > F ^1^
Extracting solvent	ChCl/Gly	156.43 ± 85.52 ^a^		<0.0001	374.65 ± 47.21 ^a^		<0.0001
	Water	93.01 ± 37.53 ^b^			211.63 ± 56.67 ^b^		
Storage conditions	Illumination	101.08 ± 59.05 ^d^		<0.0001	279.73 ± 106.0 ^c^		<0.0001
	Lack of light	135.47 ± 80.89 ^b^			292.44 ± 97.50 ^b^		
	Refrigerated	140.23 ± 80.14 ^a^			307.60 ± 89.95 ^a^		
	Environment	121.91 ± 67.82 ^c^			292.79 ± 98.05 ^b^		
Extracting solvent * Storage conditions		CHCl:Gly solvent	Water solvent	<0.0001	CHCl:Gly solvent	Water solvent	<0.0001
	Illumination	114.81 ± 73.28 ^A,d^	87.35 ± 38.84 ^B,c^		365.29 ± 63.47 ^A,d^	194.18 ± 59.01 ^B,d^	
	Lack of light	172.43 ± 93.84 ^A,b^	98.50 ± 43.50 ^B,a^		367.65 ± 55.94 ^A,c^	217.23 ± 66.36 ^B,b^	
	Refrigerated	188.93 ± 84.89 ^A,a^	92.31 ± 35.19 ^B,ab^		389.41 ± 27.49 ^A,a^	225.96 ± 40.26 ^B,a^	
	Environment	149.93 ± 81.20 ^A,c^	93.88 ± 36.19 ^B,ab^		376.41 ± 35.00 ^A,b^	209.16 ± 60.13 ^B,c^	

^1^ Significant probabilities (*p* < 0.05) by F test. For independent factors, means followed by different lowercase letters in the column differ using the t or Tukey’s tests (*p* < 0.05). In the interaction, for the same storage condition, in the row, means followed by different capital letters differ from each other using the *t*-test (*p* < 0.05). The means followed by different lowercase letters in the column for the same extracting solvent differ using Tukey’s test (*p* < 0.05).

## Data Availability

Data are contained within the article.
